# *Nymphoides peltata* Alleviates Patulin-Induced Glutamine Metabolic Stress and Epithelial Toxicity in Small Intestinal Epithelial Cells

**DOI:** 10.3390/toxins17070337

**Published:** 2025-07-03

**Authors:** Chae Hyun Lee, Sangsu Shin, Tae Hyun Kim, Sang In Lee

**Affiliations:** 1 Department of Animal Science and Biotechnology, Kyungpook National University, Sangju 37224, Republic of Korea; dlcogus9602@naver.com (C.H.L.); sss@knu.ac.kr (S.S.); 2 Research Institute for Innovative Animal Science, Kyungpook National University, Sangju 37224, Republic of Korea; 3 Department of Animal Science, The Pennsylvania State University, University Park, PA 16802, USA

**Keywords:** patulin, glutamine metabolism, ER stress, inflammation, intestinal epithelial barrier, natural products, *Nymphoides peltata*

## Abstract

Patulin (PAT) is a mycotoxin commonly found in fruits and contaminated feedstuffs, known for its gastrointestinal and systemic toxicity. However, the mechanisms underlying PAT-induced damage to intestinal epithelial cells remain poorly understood. In this study, we demonstrated that 6.5 µM PAT exposure for 24 h reduced glutamine (GLN) uptake and altered the expression of GLN transporters and related metabolic enzymes in IPEC-J2 cells. This concentration was selected based on previous in vitro studies that reported PAT-induced cytotoxicity in porcine intestinal epithelial cells. Moreover, PAT also upregulated ER stress markers (*DDIT3*, *EIF2AK3*, *ERN1*, and *HSPA5*) and inflammatory cytokines (*IL-8*, *IL-1β*, and *TNF-α*), while decreasing ZO-1 localization, indicating disrupted epithelial barrier integrity. Although 6 mM GLN supplementation only partially mediated ER stress and inflammatory responses, it more effectively restored ZO-1 localization. A high-throughput screening of 324 natural products was conducted to identify potential protective agents, identifying *Nymphoides peltata* extract as a promising candidate. Co-treatment with 80 ng/μL *N. peltata* extract improved GLN uptake, partially alleviated ER stress and inflammation, and significantly restored tight junction structure in PAT-exposed cells. Collectively, these findings suggest that *N. peltata* could serve as a novel natural therapeutic for enhancing intestinal resilience against PAT-induced toxicity. Specifically, this study highlights the potential use of *N. peltata* extract as a natural feed additive to protect intestinal health in livestock under mycotoxin stress.

## 1. Introduction

Patulin (PAT), a mycotoxin produced by various species of *Penicillium* and *Aspergillus*, has been identified as a major contaminant of fruits and fruit-derived products, particularly apples and apple juices. In addition to fruits, PAT contamination has been reported in a variety of other food commodities, including vegetables and cereals, as well as mold-damaged produce, raising significant food safety and public health concerns. According to previous studies, PAT exhibits systemic toxicity, with the kidneys, liver, gastrointestinal tract, and immune system being primary targets [1,2,3]. Furthermore, PAT is known for its electrophilic and thiol-reactive properties, forming covalent bonds with sulfhydryl groups in proteins, amino acids, and other essential biomolecules in vitro [4,5]. However, in vivo studies have shown inconsistent toxicological outcomes, likely due to differences in the exposure dose, metabolic processing, and antioxidant defense systems [6]. Despite these known properties, the molecular mechanisms through which PAT damages intestinal epithelial cells remain unclear.

After ingesting mycotoxin-contaminated food or feed, the gastrointestinal tract becomes the primary site exposed to their potentially harmful effects [7]. Upon oral exposure, PAT primarily targets the intestinal tract, as demonstrated in several experimental models. For instance, oral administration of PAT (100 μg/kg) for three consecutive days in Wistar rats induced neutrophil infiltration and villus degeneration in the small intestine [8], while 800 μg/kg PAT exposure in C57BL/6J mice resulted in severe villous damage and lamina propria inflammation [9]. In vitro studies have similarly shown that PAT (50 μM) reduces transepithelial electrical resistance and cell viability in Caco-2 cells, indicating impaired barrier function and epithelial integrity [10].

Glutamine (GLN), the most abundant free α-amino acid in the body, is widely present in plasma, skeletal muscle, milk, and fetal fluids and plays an essential role in maintaining both systemic and intestinal health [11,12,13]. In the intestines, it supports epithelial cell proliferation and contributes to the repair and maintenance of barrier integrity [14,15]. Although GLN is not a direct antioxidant, it contributes to cellular redox balance by fueling the biosynthesis of glutathione (GSH) and NADPH, which are essential for maintaining oxidative homeostasis and protecting against cellular stress [16,17,18]. In vivo and in vitro studies have demonstrated that GLN supplementation alleviates intestinal atrophy, improves nutrient absorption, and enhances tight junction protein expression, particularly during stress-inducing periods such as weaning or pathogenic assault [19,20,21]. Despite the extensive evidence supporting its beneficial effects, the specific mechanisms by which GLN counteracts the toxicity of toxins such as PAT toward intestinal epithelial cells are still not fully understood and require further investigation.

Recently, natural products have emerged as valuable sources of therapeutic agents due to the structural diversity of their chemical constituents and their wide range of biological activities [22,23]. Numerous plant-derived compounds have been reported to possess anti-inflammatory, antioxidant, and epithelial-protective properties, making them promising candidates for the prevention and treatment of intestinal disorders [24,25]. These beneficial effects may arise through direct mechanisms, such as scavenging of reactive oxygen species (ROS), or indirect mechanisms, including the upregulation of endogenous antioxidant systems like glutathione (GSH) synthesis and the modulation of inflammatory signaling pathways [26,27]. However, despite their therapeutic potential, few studies have specifically explored whether natural products can restore GLN homeostasis and alleviate PAT-induced intestinal epithelial damage. Given an increasing interest in dietary and pharmacological interventions aimed at maintaining gut health under toxin stress, identifying natural compounds capable of alleviating PAT-associated metabolic disruption represents an important avenue of research.

Therefore, we hypothesized that patulin exposure would impair glutamine metabolism and induce ER stress, inflammation, and epithelial barrier dysfunction in porcine small intestinal epithelial cells. Furthermore, we proposed that plant-derived compounds, rich in antioxidant and anti-inflammatory phytochemicals, may ameliorate these effects by restoring glutamine uptake and tight junction integrity.

## 2. Results

### 2.1. PAT Suppresses GLN Uptake and Alters GLN Transporter Expression

**Figure 1:**
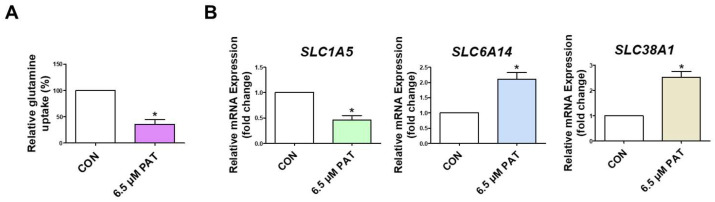
PAT reduces GLN uptake and alters transporter expression in IPEC-J2 cells. (**A**) The GLN uptake was measured using a glutamine assay kit after the 6.5 µM PAT treatment. (**B**) The relative mRNA expression of *SLC1A5*, *SLC6A14*, and *SLC38A1* was assessed by qPCR. The error bars indicate the standard error of three replicates. Statistically significant differences between the means are denoted by asterisks: *, *p* < 0.05.

To determine whether PAT affects GLN absorption in intestinal epithelial cells, a GLN uptake assay was performed in IPEC-J2 cells incubated with or without 6.5 µM PAT for 24 h. Uptake was significantly lower in PAT-treated cells than in the control group cells (Figure 1A). Additionally, the expression levels of GLN transporters were analyzed using qPCR. The PAT treatment markedly reduced the expression of *SLC1A5*, while it increased the expression levels of *SLC6A14* and *SLC38A1* (Figure 1B).

### 2.2. PAT Induces GLN Synthetase and Glutaminase Activity

**Figure 2:**
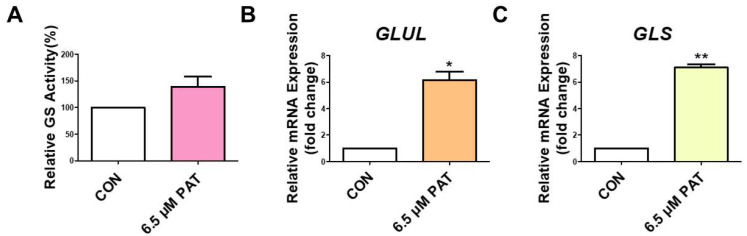
PAT upregulates GLN synthetase and glutaminase activity in IPEC-J2 cells. (**A**) The GLN synthetase activity was determined using a GLN synthetase activity assay kit. (**B**,**C**) *GLUL* and *GLS* mRNA levels were measured using qPCR. The error bars indicate the standard error of three replicates. Statistically significant differences between the means are denoted by asterisks: *, *p* < 0.05; **, *p* < 0.01.

To investigate the effect of PAT on GLN metabolism, the activity of GLN synthetase was measured. 6.5 µM PAT increased GLN synthetase activity compared to the control (Figure 2A). Subsequently, the gene expression of *GLUL* (glutamine synthetase) and *GLS* (glutaminase) was evaluated using qPCR. Both *GLUL* and *GLS* mRNA levels were significantly elevated following the PAT treatment (Figure 2B,C).

### 2.3. PAT Activates ER Stress and Inflammatory Cytokine Expression

**Figure 3:**
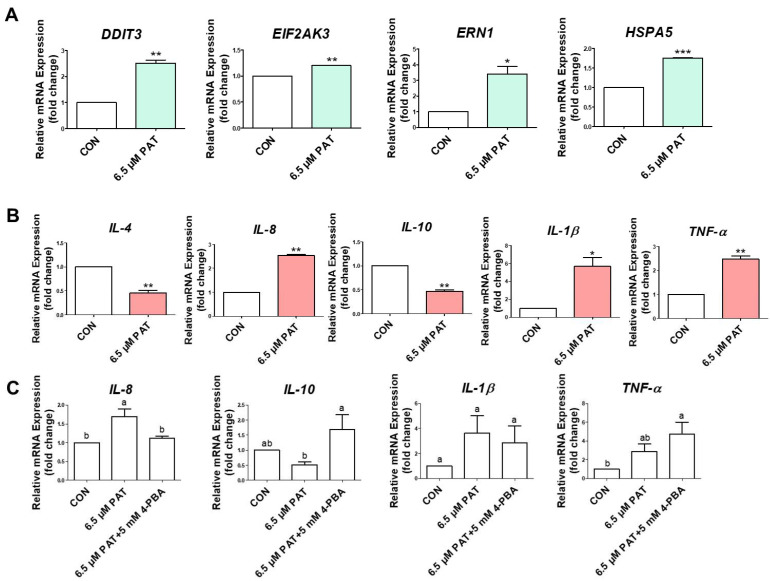
PAT induces ER stress and inflammatory cytokine expression. (**A**) The expression of the ER stress-related genes *DDIT3*, *EIF2AK3*, *ERN1*, and *HSPA5* was elevated in the PAT group. (**B**) The expression of the pro- and anti-inflammatory cytokine-encoding genes *IL-4*, *IL-8*, *IL-10*, *IL-1β*, and *TNF-α* was determined using qPCR. Statistically significant differences between the means are denoted by asterisks: *, *p* < 0.05; **, *p* < 0.01; and ***, *p* < 0.001. (**C**) The 5 mM 4-PBA treatment attenuated PAT-induced changes in cytokine expression. The lowercase letters (“a” and “b”) indicate statistically significant differences between the treatments based on Duncan’s multiple range test. The error bars indicate the standard error of three replicates.

To assess ER stress, qPCR was performed to measure the expression levels of *DDIT3*, *EIF2AK3*, *ERN1*, and *HSPA5*. The expression of all markers was significantly greater in the 6.5 µM PAT-treated cells than in the control group cells (Figure 3A). The expression of the inflammatory cytokine genes *IL-4*, *IL-8*, *IL-10*, *IL-1β*, and *TNF-α* was also evaluated. Patulin treatment significantly increased the expression of *IL-8*, *IL-1β*, and *TNF-α*, while significantly decreasing the expression of *IL-4* and *IL-10* (Figure 3B). Treatment with the ER stress inhibitor 4-PBA in combination with PAT selectively modulated the expression of inflammatory cytokines in IPEC-J2 cells. The expression of *IL-8* was significantly decreased following the 5 mM 4-PBA treatment, while *IL-10* expression was significantly increased compared to that of the PAT-only group. For *IL-1β*, expression was reduced by the 4-PBA treatment, although the change was not statistically significant, and *TNF-α* expression was further elevated over that of the PAT-only treatment (Figure 3C).

### 2.4. PAT Disrupts Epithelial Barrier Integrity

**Figure 4:**
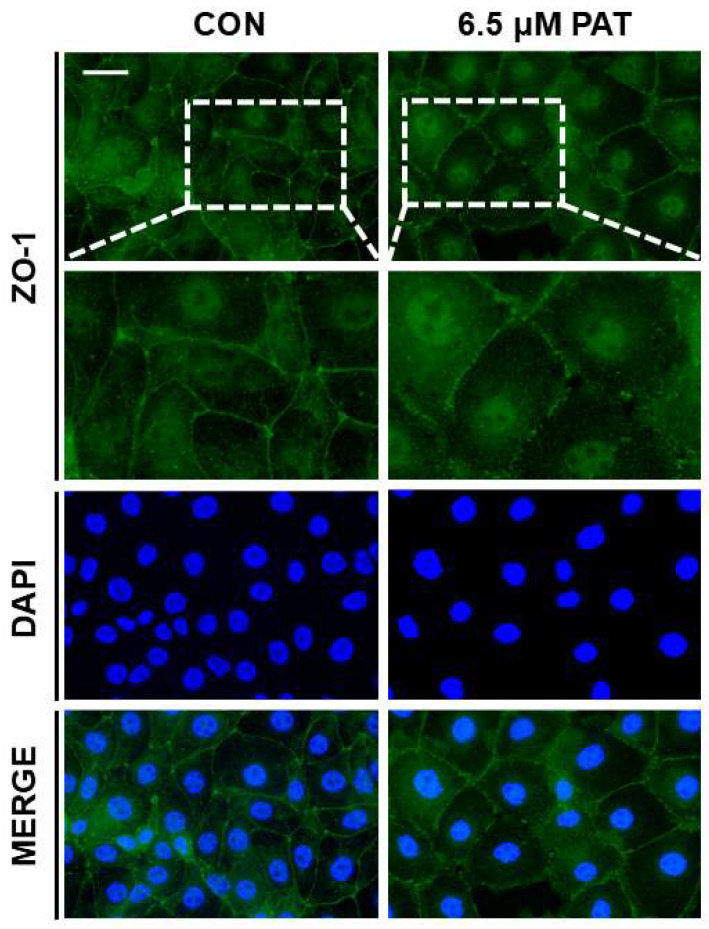
PAT disrupts tight junction integrity in IPEC-J2 cells. The immunofluorescence staining of ZO-1 (green) showed continuous localization at the cell borders in the control cells, while the 6.5 µM PAT-treated cells exhibited discontinuous and fragmented ZO-1 distribution (scale bar = 40 μm). The nuclei were stained with 4′,6-diamidino-2-phenylindole (DAPI; blue).

To evaluate the impact of PAT on intestinal barrier function, immunofluorescence staining was performed to assess the localization of the tight junction protein ZO-1. In the control cells, ZO-1 was continuously distributed along the cell borders, reflecting intact tight junctions. In contrast, treatment with 6.5 µM PAT resulted in a disrupted and fragmented ZO-1 staining pattern, indicative of impaired epithelial barrier integrity (Figure 4).

### 2.5. Effects of GLN on PAT-Induced ER Stress, Inflammatory Responses, and Barrier Dysfunction in IPEC-J2 Cells

**Figure 5:**
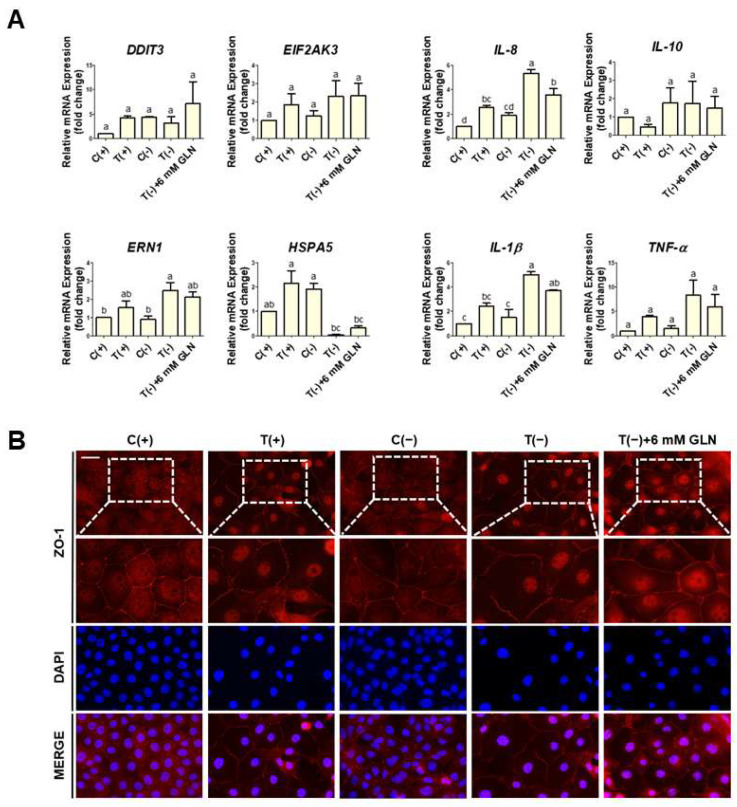
GLN supplementation alleviates PAT-induced ER stress, inflammation, and barrier dysfunction. IPEC-J2 cells were cultured under five experimental conditions: C(+), control cells cultured in a GLN-containing medium; T(+), 6.5 µM PAT-treated cells cultured in a GLN-containing medium; C(−), control cells cultured in a GLN-free medium; T(−), 6.5 µM PAT-treated cells cultured in a GLN-free medium; and T(−)+6 mM GLN, 6.5 µM PAT-treated cells in a GLN-free medium supplemented with 6mM GLN. (**A**) The expression of the ER stress-related genes *DDIT3*, *EIF2AK3*, *ERN1*, and *HSPA5* and the inflammatory cytokine genes *IL-8*, *IL-10*, *IL-1β*, and *TNF-α* were measured using qPCR. The lowercase letters (“a”, “b”, “c”, and “d”) denote statistical differences between the experimental groups. The error bars represent the standard errors of three independent experiments. (**B**) The immunocytochemical analysis of ZO-1 (red) in IPEC-J2 cells under different treatment conditions is shown (scale bar = 40 μm). In the control cells, ZO-1 showed a continuous, linear staining pattern along the cell borders, indicative of intact tight junction organization. In contrast, the 6.5 µM PAT-treated cells exhibited discontinuous and fragmented ZO-1 localization, especially under GLN-deprived conditions, suggesting junctional disorganization. GLN supplementation (6 mM) appeared to improve the continuity of ZO-1 membrane localization in PAT-treated, GLN-deficient group cells. The nuclei were counterstained with 4′,6-diamidino-2-phenylindole (DAPI; blue).

To determine whether GLN supplementation could mediate PAT-induced ER stress and inflammatory responses, IPEC-J2 cells were cultured in five media: a control with GLN (C(+)), 6.5 µM PAT with GLN (T(+)), a control without GLN (C(−)), 6.5 µM PAT without GLN (T(−)), and 6.5 µM PAT with 6 mmol/L, a high-dose, of GLN (T(−)+6 mM GLN). Among the four ER stress-related genes examined, the *ERN1* expression exhibited a mild decrease when 6mM GLN was added to the T(−) media compared to the T(−) group, while those of *DDIT3*, *EIF2AK3*, and *HSPA5* were similar to the T(−) group. Although 6mM GLN did not markedly reverse the effects of PAT on the expression of most ER stress markers, the partial modulation of *ERN1* expression suggests that GLN may selectively influence specific branches of the ER stress response. Similarly, 6 mM GLN supplementation decreased the expression of all inflammatory marker genes compared to the T(−) media group (Figure 5A). To evaluate whether GLN could mitigate PAT-induced epithelial barrier dysfunction, the immunofluorescence staining of ZO-1, a tight junction protein, was performed on IPEC-J2 cells cultured under five different conditions. In the C(+) group, ZO-1 was continuously distributed along the cell borders, indicating intact tight junctions. In contrast, the cells of both 6.5 µM PAT-treated groups, T(+) and T(−), exhibited discontinuous or fragmented membrane-associated ZO-1 staining, particularly in T(−) group cells, suggesting substantial disruption of junctional organization. Notably, supplementation with 6 mM of GLN restored a more continuous junctional pattern compared to the T(−) group (Figure 5B).

### 2.6. Protective Natural Products Showing Promise in Counteracting PAT-Induced Cytotoxicity

**Figure 6:**
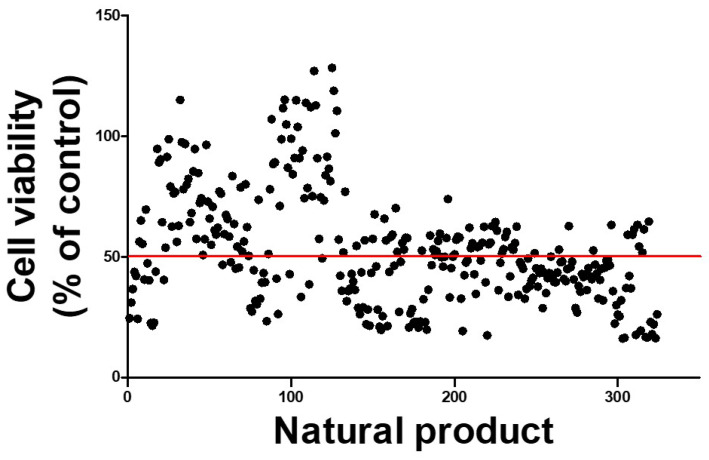
High-throughput screening of natural products assessing their protective effects against PAT-induced cytotoxicity in IPEC-J2 cells. To identify candidate natural products capable of alleviating PAT toxicity, IPEC-J2 cells were co-treated with 6.5 μM (approximately 1.00 ng/µL) PAT and 20 ng/μL of each natural product for 24 h. The cell viability was then quantified and plotted as a percentage relative to the cell viability of untreated control cells. Each black dot on the scatter plot represents the viability outcome of a single natural product treatment. The red horizontal line denotes the IC_50_ threshold for the PAT-treated cells, serving as a reference point to identify the compounds that conferred increased cell survival.

To discover natural products with the potential to alleviate PAT’s toxic effects, we conducted a high-throughput screening assay using a library of 324 plant-derived compounds. An amount of 20 ng/μL of each compound was co-administered with 6.5 µM PAT to IPEC-J2 cells, and the cell viability was assessed as a simple measure of each product’s cytoprotective effect. While most candidates exhibited no significant protective effect or even exacerbated cytotoxicity, a subset of compounds demonstrated improved cell survival compared to that under the PAT treatment (IC_50_ reference level). Among them, a few compounds notably increased cell viability, with the top three exhibiting viability rates exceeding 110% relative to the untreated control (Figure 6). We selected *Nymphoides peltata* extract as the most efficient natural product among them.

### 2.7. Nymphoides peltata Extract Modulates GLN Uptake, ER Stress, Inflammation, and Tight Junction Localization in PAT-Treated IPEC-J2 Cells

**Figure 7:**
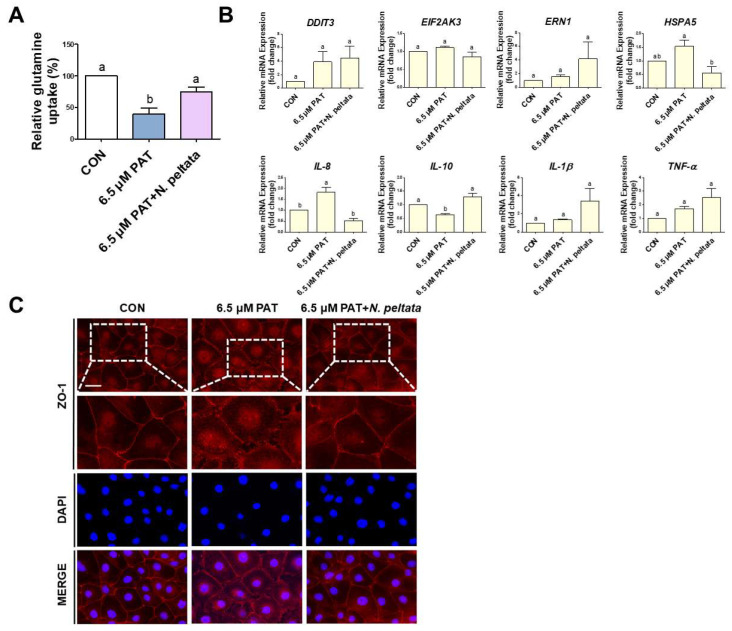
The effects of *N. peltata* extract on GLN uptake, ER stress, inflammatory markers, and ZO-1 expression in PAT-treated IPEC-J2 cells. (**A**) GLN uptake was assessed following treatment with 6.5 µM PAT alone or in combination with 80 ng/μL of *N. peltata* extract. While 6.5 µM PAT significantly reduced GLN uptake, co-treatment with 80 ng/μL *N. peltata* restored uptake levels toward those of the control. (**B**) The expression of the ER stress-related genes *DDIT3*, *EIF2AK3*, *ERN1*, and *HSPA5* and the inflammatory cytokine-encoding genes *IL-8*, *IL-10*, *IL-1β*, and *TNF-α* was evaluated using qPCR. Treatment with 80 ng/μL *N. peltata* partially restored the expression of *EIF2AK3*, *HSPA5*, *IL-8*, and *IL-10* to normal (control) levels, whereas the expression of *DDIT3*, *ERN1*, *IL-1β*, and *TNF-α* remained elevated or were further increased. The lowercase letters (“a” and “b”) denote statistical differences between the control and the treatment groups. The error bars represent the standard error of three independent experiments. (**C**) The immunofluorescence staining of ZO-1 (red) and DAPI (blue) was performed to assess epithelial barrier integrity (scale bar = 40 μm). The 6.5 µM PAT treatment disrupted the continuous membrane localization of ZO-1, while co-treatment with 80 ng/μL *N. peltata* extract partially restored its junctional distribution.

To further evaluate the potential protective effects of the *N. peltata* extract, co-treatment experiments with PAT were conducted using IPEC-J2 cells. The co-treatment with 80 ng/μL *N. peltata* increased GLN uptake compared to the 6.5 µM PAT-only group (Figure 7A). The expression levels of the ER stress-related genes *DDIT3*, *EIF2AK3*, *ERN1*, and *HSPA5* and the inflammatory cytokine-encoding genes *IL-8*, *IL-10*, *IL-1β*, and *TNF-α* were then examined. The *N. peltata* extract treatment resulted in the partial modulation of ER stress and inflammatory markers. Specifically, *EIF2AK3*, *HSPA5*, *IL-8*, and *IL-10* all, at least partially, returned to normal (control) levels when the extract was included along with 6.5 µM PAT in the media, suggesting a partial alleviation of ER stress and inflammation (Figure 7B). Finally, ZO-1 immunofluorescence staining was performed to assess epithelial barrier integrity. The 6.5 µM PAT treatment markedly affected the ZO-1 distribution, disrupting the continuous staining along the cell borders, but co-treatment with 80 ng/μL *N. peltata* improved ZO-1 localization at cell–cell junctions (Figure 7C). This suggests that *N. peltata* may help maintain or restore tight junction integrity in the context of PAT-induced epithelial damage.

### 2.8. The Identification of the Abundant Bioactive Compounds in N. peltata Extract Using LC-MS/MS

**Figure 8:**
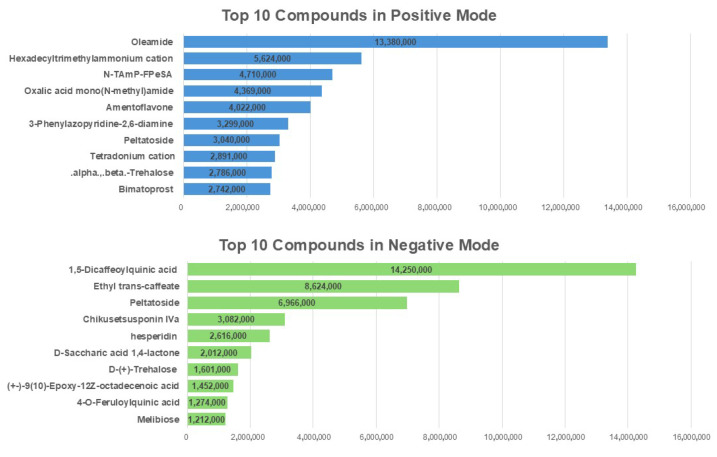
The top 10 most abundant compounds identified in the 20 mg/mL *N. peltata* extract using LC-MS/MS in positive and negative ionization modes. The compounds were sorted in descending order based on their peak areas, and the bars indicate their relative abundances.

To explore the bioactive constituents of the 20 mg/mL *N. peltata* extract and identify those that may be responsible for its protective effects, a liquid chromatography–tandem mass spectrometry (LC-MS/MS) analysis was performed, using both positive and negative ionization modes to obtain a comprehensive profile of the extract’s chemical composition. A total ion chromatogram was generated, and compounds were ranked based on their peak areas, which estimate their relative abundances. The top 10 most abundant compounds were identified and are listed in descending order in Figure 8.

## 3. Discussion

Patulin is a mycotoxin primarily produced by *Penicillium* and *Aspergillus* species, frequently contaminating fruits, especially apples and apple-derived products [28]. Due to its widespread occurrence in the food and feed supply chain, PAT poses significant risks to both human and animal health [29]. Numerous studies have shown that PAT induces systemic toxicity, particularly affecting the gastrointestinal tract, where it compromises epithelial integrity, induces inflammation, and interferes with nutrient absorption [30,31]. However, the underlying molecular mechanisms, especially those at work in intestinal epithelial cells, are not well-understood. In this study, we used 6.5 μM PAT, a concentration consistent with previous in vitro studies reporting cytotoxic effects in porcine intestinal epithelial cells [32]. Although in vivo exposure levels may vary depending on species, dose, and duration, this concentration provides a physiologically relevant model to investigate the cellular mechanisms of PAT toxicity and to screen potential protective compounds.

Given the importance of GLN in maintaining epithelial health, barrier function, and cellular metabolism, we hypothesized that PAT-induced toxicity may involve GLN metabolism disruption. Furthermore, we aimed to explore whether GLN supplementation or natural product-based interventions could attenuate these toxic effects. Building on our previous work investigating the abilities of natural products to mitigate PAT toxicity, the present study was designed to clarify the role of GLN metabolism in PAT-induced epithelial dysfunction and identify bioactive plant compounds capable of offering protection.

We found that 6.5 μM PAT decreased the gene expression of *SLC1A5*, while *SLC6A14* and *SLC38A1* mRNA levels increased. Glutamine uptake is regulated by *SLC1A5*, and loss of *SLC1A5* function inhibits cell growth and activates autophagy [33]. The decreased expression of *SLC1A5* in this study is consistent with the results of a previous study in which Myc haploinsufficiency was found to be accompanied by decreased *SLC1A5* expression and decreased glutamine uptake and metabolism in mouse liver tissue, tail fibroblasts, and hepatocytes. In addition, this finding is also consistent with a previous study in which mice exposed to zearalenone (ZEA), another type of mycotoxin, showed downregulation of *SLC1A5* mRNA expression in the placenta, as determined by quantitative PCR, using ZEA doses of 0, 2.5, 5, 10, and 20 mg/kg, compared to the control group [34]. As a broad-specificity amino acid transporter, *SLC6A14* can mediate the uptake of GLN and other neutral amino acids, and its expression has been shown to increase under metabolic stress or when canonical GLN transporters are downregulated [35]. Similarly, *SLC38A1* is upregulated in various cancer types and has been implicated in supporting cell survival and proliferation when GLN availability is limited [36].

One of the most intriguing findings in this study was the concurrent upregulation of both glutamine synthetase (*GLUL*) and glutaminase (*GLS*) in PAT-treated IPEC-J2 cells, despite a significant reduction in GLN uptake. This observation suggests a profound metabolic shift, likely initiated to compensate for the scarcity of external GLN and increased intracellular demand. In a previous study, *GLUL* expression was significantly upregulated during GLN deprivation in a sarcoma model, peaking between 48 and 72 h after stress exposure, and this upregulation allowed cells to adapt and eventually resume proliferation after an initial growth arrest. However, the study highlighted the fact that while GS activity is essential, it is not sufficient to fully compensate for GLN loss [37], suggesting that increased GS may not fully compensate for the loss of GLN. This is consistent with our observation that increased *GLUL* expression under PAT-induced stress did not restore GLN homeostasis. The upregulation of *GLS* suggests a complementary, but potentially counterproductive, adaptive mechanism. Glutaminase converts GLN to glutamate, fueling the TCA cycle and supporting energy production and biosynthesis. Recent studies in pancreatic cancer cells have shown that *GLS* expression is induced in response to nutrient deprivation, and that *GLS* hyperactivation promotes metabolic reprogramming and cancer progression [38]. Our data may reflect a similar mechanism, whereby PAT-treated epithelial cells upregulate *GLS* in an effort to sustain themselves, despite the risk of accelerating intracellular GLN depletion. Ultimately, this dysregulation may worsen GLN depletion and exacerbate downstream cellular stress, including ER stress, inflammation, and barrier dysfunction.

As this suggests, we found that the expression of the endoplasmic reticulum (ER) stress markers *DDIT3*, *EIF2AK3*, *ERN1*, and *HSPA5* and the inflammatory cytokine-encoding genes *IL-8*, *IL-1β*, and *TNF-α*, was significantly upregulated by 6.5 μM PAT (24 h), while the anti-inflammatory cytokines *IL-4* and *IL-10* were downregulated. However, the relationship between ER stress and inflammation proved to be marker-specific and limited. While treatment with the ER stress inhibitor 5 mM 4-PBA restored *IL-10* expression and significantly reduced *IL-8* expression, *TNF-α* expression paradoxically increased, and the expression of *IL-1β* showed only a modest, statistically insignificant reduction. These findings suggest that ER stress partially contributes to—but does not fully explain—the inflammatory responses triggered by PAT, implying the involvement of parallel or independent inflammatory pathways.

Although GLN supplementation is known to alleviate ER stress or inflammation under various stress conditions (thioacetamide, ischemia-reperfusion injury, and lipopolysaccharide) [39,40,41], there is no study on the alleviation of ER stress and inflammation increased by patulin treatment. In our model, 6 mM GLN supplementation failed to significantly reverse the expression of major ER stress genes, such as *DDIT3* and HSPA5, while only *ERN1* expression was modestly reduced. Likewise, the anti-inflammatory effects of GLN were selective. *IL-8* expression was significantly reduced by GLN, while *IL-10* expression remained unaffected, and *TNF-α* and *IL-1β* showed only modest, non-significant decreases. These findings imply that GLN preferentially targets chemokine-mediated epithelial stress responses (e.g., *IL-8*), rather than broad immunosuppressive pathways such as *IL-10* signaling. Taken together, our results reveal that GLN does not uniformly inhibit ER stress or inflammation in PAT-exposed cells, but rather acts in a pathway- and marker-dependent manner. These findings underline the need for future studies to delineate the precise molecular interactions between GLN metabolism, ER stress sensors, and downstream immune signaling cascades under mycotoxin-induced stress conditions.

Zonula occludens-1 is a key scaffolding protein that plays a critical role in maintaining epithelial tight junction integrity by linking transmembrane tight junction proteins to the actin cytoskeleton and regulating paracellular permeability [42,43]. The loss or disruption of ZO-1 is commonly associated with impaired barrier function, increased intestinal permeability, and heightened susceptibility to inflammatory injury [44,45]. In our study, immunofluorescence assays revealed that 6.5 μM PAT exposure disrupted the typical membrane-associated localization of ZO-1, resulting in a discontinuous pattern at the cell borders, indicative of tight junction disassembly and compromised epithelial barrier integrity. However, 6 mM GLN supplementation significantly restored ZO-1 distribution at cell–cell contact sites. These results are consistent with a previous study in which 2 mM GLN supplementation significantly restored the expression of tight junction-related genes (ZO-1, occludin, and claudin-3) in IPEC-J2 cells exposed to 20 μg/mL zearalenone, another mycotoxin known to impair barrier function [46]. Similarly, in our study, GLN supplementation mitigated the PAT-induced disruption of ZO-1 localization at the cell membrane. Therefore, while GLN exhibited limited efficacy in reversing ER stress and inflammatory marker expression, it showed robust protective effects on epithelial structural integrity, demonstrating its importance in preserving barrier function under mycotoxin-induced stress conditions.

*Nymphoides peltata*, commonly known as the yellow floating heart, is an aquatic perennial plant traditionally used in herbal medicine. Members of the genus *Nymphoides* are rich in polyphenols, flavonoids, triterpenes, and ferulic acid, and are recognized for their pharmacological properties, which include antioxidant, anti-diabetic, and anticonvulsant activities [47,48]. The LC-MS/MS analysis of the *N. peltata* extract used in our study revealed a rich phytochemical profile, including 1,5-dicaffeoylquinic acid, hesperidin, oleamide, and amentoflavone, compounds widely recognized for their antioxidant, anti-inflammatory, and cytoprotective effects in previous studies [49,50,51,52]. Given that PAT toxicity is closely associated with GLN metabolic stress, oxidative stress, ER stress, and epithelial barrier dysfunction, these phytochemicals may contribute to the extract’s ability to mitigate the toxic effects of PAT. Although LC-MS/MS analysis enabled the tentative identification of the major compounds present in *N. peltata* extract, it should be noted that this method alone is insufficient for definitive structural characterization. Moreover, compounds with high peak areas in LC-MS/MS do not necessarily contribute to the observed protective effects. Therefore, additional fractionation and target-based validation will be required to identify the true bioactive constituents. The precise identification and structural elucidation of these compounds require further isolation and spectroscopic analyses, such as NMR and MS/MS. Future studies are therefore warranted to isolate these abundant compounds and investigate their individual and combined effects on GLN uptake and tight junction protein expression in PAT-treated IPEC-J2 cells. We found that co-treatment with 80 ng/μL *N. peltata* extract partially restored GLN uptake, modulated ER stress- and inflammation-associated gene expression, and significantly enhanced ZO-1 localization in 6.5 μM PAT-exposed IPEC-J2 cells. Collectively, these findings identify *N. peltata* as a promising natural product for further development as a functional feed additive aimed at enhancing intestinal barrier function and mitigating PAT-induced toxicity. Moreover, recent studies in pig models have demonstrated that essential oils and hydrolyzed protein formulas can enhance antioxidant capacity, improve nutrient digestion and absorption, and promote gut health and microbial balance, highlighting the potential of natural dietary strategies to support intestinal function under pathological or stress conditions [53,54]. Ultimately, this research contributes to a growing body of evidence supporting the use of natural product-based strategies to enhance intestinal resilience against mycotoxins. This approach may provide a sustainable, non-antibiotic alternative approach to improving gut health and increasing productivity in livestock production.

## 4. Conclusions

This study investigated the protective effects of *N. peltata* extract against PAT-induced toxicity in porcine intestinal epithelial cells. We showed that 6.5 μM PAT exposure disrupts GLN metabolism, induces ER stress and inflammatory responses, and impairs epithelial barrier integrity. Glutamine supplementation at 6 mM partially alleviated ER stress and inflammation and significantly improved the localization of ZO-1 at cell–cell junctions. Additionally, 80 ng/μL *N. peltata* improved GLN uptake, modulated stress responses, and enhanced epithelial barrier function. Collectively, our findings suggest that *N. peltata* has promise as a natural feed additive for mitigating the adverse effects of PAT on pig intestinal health. This study provides new insights into the mechanisms of PAT-induced intestinal epithelial cell damage and highlights the potential of natural products as alternative strategies for improving livestock productivity and resilience.

## 5. Materials and Methods

### 5.1. Reagents

Dulbecco’s modified Eagle’s medium (DMEM), phosphate-buffered saline (PBS), fetal bovine serum (FBS), and penicillin-streptomycin were purchased from Thermo Fisher Scientific (Wilmington, DE, USA). Patulin (PAT) and glutamine were purchased from Sigma-Aldrich (St. Louis, MO, USA). 4-Phenylbutyric acid (4-PBA) was purchased from MedChemExpress (Monmouth Junction, NJ, USA). Primary antibodies specific for ZO-1 were purchased from Thermo Fisher Scientific (Wilmington, DE, USA).

### 5.2. Cell Culturing and Treatment

The IPEC-J2 cell line, derived from the jejunum of unsuckled neonatal piglets, was obtained from DSMZ (Braunschweig, Germany). The cells were cultured in Dulbecco’s modified Eagle’s medium (DMEM), supplemented with fetal bovine serum (FBS) at 10% and penicillin–streptomycin at 1%. Cultivation was carried out at 37 °C and 5% CO_2_ in a humidified incubator. For use in treatments, PAT was dissolved in dimethyl sulfoxide (DMSO) to prepare a 10 mM stock solution and stored at −20 °C. Prior to use, the stock was diluted in a culture medium to a final concentration of 6.5 µM, based on the IC_50_ value reported in our previous study [27]. The treatment of PAT in IPEC-J2 cells was set to 24 h, similar to our previous study.

### 5.3. Glutamine Uptake Assay

IPEC-J2 cells were seeded at 3 × 10^5^ cells/well in 60 pi plates and incubated for 24 h in medium. Following incubation, the cells were treated with 6.5 µM PAT for an additional 24 h. A glutamine assay kit (ab197011, Abcam, Cambridge, UK) was used to evaluate GLN uptake in accordance with the manufacturer’s instructions. Glutamine concentrations were measured in both the raw medium and the medium collected after cell incubation or treatment, and GLN consumption was calculated as the difference between these two values (i.e., GLN in raw medium − GLN in the medium after culturing/treating cells), normalized based on the total protein content of each sample. The results are expressed as the GLN uptake relative to that of the control group in each independent experiment. All data represent the mean values obtained from three separate biological replicates.

### 5.4. Glutamine Synthetase Activity Assay

IPEC-J2 cells were seeded at a density of 3 × 10^5^ cells/well in 60 pi plates and treated with 6.5 μM PAT for 24 h. Following treatment, the glutamine synthetase (GS) activity was measured using a glutamine synthetase activity assay kit (ab284572, Abcam, Cambridge, UK), according to the manufacturer’s instructions. This assay is based on the GS-mediated conversion of glutamate and ammonia to GLN in an ATP-dependent reaction, with ADP produced as a byproduct. The ADP is further processed through a series of enzymatic reactions involving the ADP Converter, ADP Developer Mix, and ADP Probe reagents, ultimately forming a colorimetric product. The absorbance at 570 nm of this product was measured using a microplate reader, and the GS activity was quantified using a standard curve and expressed as the mean activity relative to the total protein concentration in each sample, based on three independent biological experiments.

### 5.5. Quantitative Real-Time PCR

The total RNA was isolated from IPEC-J2 cells using the AccuPreP Universal RNA Extraction Kit (Cat. No. K-3141, BioNEER, Daejeon, Republic of Korea), according to the manufacturer’s protocol. Subsequently, 1 μg of total RNA was reverse-transcribed into complementary DNA (cDNA) using the DiaStar™ RT Kit (SolGent, Daejeon, Republic of Korea). For the qPCR analysis, gene-specific primers (Table 1) were designed using the Primer3 software (http://frodo.wi.mit.edu (accessed on 30 June 2025)). The thermocycler protocol included an initial denaturation at 95 °C for 3 min, followed by 40 cycles of 95 °C for 15 s, 56–58 °C for 15 s, and 72 °C for 15 s. The relative gene expression levels were calculated using the 2−ΔΔCt method, using GAPDH as the internal control for normalization. All of the experiments were conducted in triplicate.

### 5.6. Immunofluorescence

IPEC-J2 cells were cultured on gelatin-coated glass coverslips and subjected to various 24 h treatments, including PAT, PAT combined with GLN, and PAT combined with the selected natural product. After treatment, the cells were fixed with 4% paraformaldehyde for 15 min at room temperature, followed by incubation with a blocking buffer for 1 h to prevent non-specific binding. The cells were then incubated overnight at 4 °C with anti-zonula occludens-1 (ZO-1) antibody (1:200, Thermo Fisher Scientific) diluted in an antibody buffer. After three washes with phosphate-buffered saline (PBS), the cells were incubated for 1 h at room temperature in the dark with Alexa Fluor ^®^ 488-conjugated goat anti-rabbit IgG H&L or Alexa Fluor ^®^ 594-conjugated goat anti-rabbit IgG H&L, both at 1:500 dilutions (Abcam, Cambridge, UK). The nuclei were counterstained with DAPI (Vector Laboratories, Burlingame, CA, USA), and the cells were covered with coverslips. Fluorescence images were captured using a fluorescence microscope (Korea Labtech, Seongnam-si, Republic of Korea).

### 5.7. High-Throughput Screening of Natural Products

A high-throughput screening analysis was conducted using a library of 324 natural products, each obtained by 70% ethanol extraction, provided by the Nakdonggang National Institute of Biological Resources (Sangju, Republic of Korea). Each natural product was initially supplied at a concentration of 20 mg/mL. IPEC-J2 cells were seeded in 96-well plates at a density of 1 × 10^4^ cells per well and incubated for 24 h. The cells were then co-treated with 6.5 μM PAT and each natural product, respectively, at a final concentration of 20 ng/μL for 24 h. Following treatment, 10 μL of EZ-Cytox (Roche Diagnostics GmbH, Mannheim, Germany) was added to each well, and the cells were incubated for 2 h at 37 °C. Cell viability was then evaluated by measuring absorbance in the 450–600 nm range using a GloMax Discover Multi-Microplate Reader (Promega, Madison, WI, USA). The list of the top 324 natural products is provided in Table S1.

### 5.8. LC-MS/MS Analysis of Nymphoides peltata Extract

The chemical composition of the *Nymphoides peltata* extract was analyzed using a liquid chromatography–tandem mass spectrometry (LC-MS/MS) system. Chromatographic separation was performed on a SHIMADZU LC-20AR system equipped with a reverse-phase C18 column (Agilent Zorbax Bonus-RP, 2.1 mm × 150 mm, 3.5 μm). The mobile phases consisted of 0.1% formic acid in water (solvent A) and 0.1% formic acid in acetonitrile (solvent B), delivered at a flow rate of 0.3 mL/min under the following gradient program: 0 min, 95% A/5% B; 30 min, 5% A/95% B; 35 min, 95% A/5% B; and maintained until 40 min. The column temperature was maintained at 40 °C, and the injection volume was set at 10 μL. Mass spectrometric detection was carried out using an AB SCIEX 500R QTOF LC-MS/MS instrument equipped with an electrospray ionization (ESI) source operated in both positive and negative ionization modes. Source parameters were set as follows: ion source gas 1 and gas 2 at 50 psi, curtain gas at 25 psi, collision-activated dissociation (CAD) gas at 7, and source temperature at 450 °C. The spray voltage was ±4500 V, with a declustering potential of 80 V and collision energy of 10 V. Data acquisition was performed in full scan mode, and compound identification was based on accurate mass measurement, retention time, and library matching using standard MS/MS databases.

### 5.9. Statistics

All experiments were performed as biologically independent triplicates, and statistical analyses were conducted using GraphPad Prism version 5.00 (GraphPad Software, Inc., La Jolla, CA, USA). The data are presented as the means ± standard errors. For comparisons between two experimental groups, a *t*-test was used [55]. For comparisons among multiple groups, one-way analyses of variance (ANOVAs), followed by Duncan’s multiple range tests, were performed [56]. A *p*-value of less than 0.05 was considered statistically significant.

## Figures and Tables

**Table 1:** The primers used for the quantitative polymerase chain reaction.

Target Gene ^a^	Sequence (5′–3′)	Accession No.
*GAPDH*	Forward: ACACCGAGCATCTCCTGACT Reverse: GACGAGGCAGGTCTCCCTAA	NM_001206359
*SLC1A5*	Forward: CTTGACTCTTGTCCCCATCC Reverse: CAACCCATCAGAACCTCCTT	XM_003127238.5
*SLC6A14*	Forward: TAACTTTGGGTTGCTGCTTG Reverse: CAATGAATCTGTTCCCTCCAT	NM_001348402.1
*SLC38A1*	Forward: CTGGGGGTGTTGTTTTCTCT Reverse: AAGGAAGTGATGAGGGGTTG	XM_003355629.4
*GLUL*	Forward: ATGCGAGAGGAGAATGGTCT Reverse: TCGTTGATGTTGGAGGTTTC	NM_213909.1
*GLS*	Forward: CTCTTGACCAGGGGTGTTCT Reverse: GGTGTTTGGGGCTGAATAGT	XM_021076049.1
*DDIT3*	Forward: TGAAAGCAGAGCCTAATCCA Reverse: CCAGGGGGTGAGACATAGTT	NM_001144845.1
*EIF2AK3*	Forward: AAAGGTCTCGGTTGCTGATT Reverse: AAAAGGCTGATGGGAATGAC	XM_003124925.4
*ERN1*	Forward: GTCTCTGCCCATCAACCTCT Reverse: ATCTTGTAGTCCCCGTCGTC	XM_005668695.3
*HSPA5*	Forward: AGGGAAGGGGAGAAGAACAT Reverse: GGTAGAACGGAAAAGGTCCA	XM_001927795.7
*IL-4*	Forward: TCCACGGACACAAGTGCGAC Reverse: TGTTTGCCATGCTGCTCAGG	NM_214123.1
*IL-8*	Forward: GGCTGTTGCCTTCTTGGCAG Reverse: TTTGGGGTGGAAAGGTGTGG	NM_213867
*IL-10*	Forward: CATCCACTTCCCAACCAGCC Reverse: CTCCCCATCACTCTCTGCCTTC	NM_214041
*IL-1β*	Forward: GAACAAGAGCATCAGGCAGA Reverse: TGGCATCACAGACAAAGTCA	NM_001305893
*TNF-α*	Forward: TTTCTGTGAAAACGGAGCTG Reverse: CAGCGATGTAGCGACAAGTT	NM_214022

^a^ Gene name abbreviations: *GAPDH*, glyceraldehyde-3-phosphate dehydrogenase; *SLC1A5*, solute carrier family 1 member 5; *SLC6A14*, solute carrier family 6 member 14; *SLC38A1*, solute carrier family 38 member 1; *GLUL*, glutamate-ammonia ligase; *GLS*, glutaminase; *DDIT3*, DNA damage inducible transcript 3; *EIF2AK3*, eukaryotic translation initiation factor 2 alpha kinase 3; *ERN1*, endoplasmic reticulum to nucleus signaling 1; *HSPA5*, heat shock protein family A (Hsp70) member 5; *IL-4*, interleukin 4; *IL-8*, interleukin-8; *IL-10*, interleukin-10; *IL-1β*, interleukin-1β; and *TNF-α*, tumor necrosis factor α.

## Data Availability

The original contributions presented in this study are included in this article/Supplementary Materials. Further inquiries can be directed to the corresponding author.

## Supplementary Materials

The following supporting information can be downloaded at: https://www.mdpi.com/article/10.3390/toxins17070337/s1, Table S1: List of top 324 natural products; Table S2: Top 10 positive and negative ion mode compounds identified from *Nymphoides peltata* extract with chemical structures.

## Abbreviations

The following abbreviations are used in this manuscript:

PAT	Patulin
GLN	Glutamine
ER	Endoplasmic reticulum
*N. peltata*	*Nymphoides peltata*
GS	Glutamine synthetase
ZO-1	Zonula occludin 1
PCR	Polymerase chain reaction
