# Supplementary material for: Nymphoides peltata Alleviates Patulin-Induced Glutamine Metabolic Stress and Epithelial Toxicity in Small Intestinal Epithelial Cells
Source: Toxins (Basel). 2025 Jul 3;17(7):337. doi: 10.3390/toxins17070337 (PMC12299487; doi:10.3390/toxins17070337)
Supplement: Supplementary file 1 [file toxins-17-00337-s001.zip › toxins-3725162-supplementary.pdf]

Table S1. List of top 324 natural products.

NO.	Natural Product	Solvent	Cell Viability(%)
1	Nymphoides peltata	70% Ethanol	128.3458528
2	Glyceria leptolepis	70% Ethanol	127.0557748
3	Nymphoides peltata	70% Ethanol	118.8934039
4	Najas marina	70% Ethanol	115.1300382
5	Scirpus radicans	70% Ethanol	115.0557893
6	Juncus diastrophanthus	70% Ethanol	114.9144597
7	Lysimachia davurica	70% Ethanol	113.8488275
8	Glyceria leptolepis	70% Ethanol	112.8614217
9	Penthorum chinense	70% Ethanol	112.048917
10	Phragmites japonica	70% Ethanol	111.6867515
11	Potamogeton malaianus	70% Ethanol	110.5587823
12	Phalaris arundinacea	70% Ethanol	107.0671738
13	Elatine triandra	70% Ethanol	104.9118368
14	Commelina communis	70% Ethanol	103.9223909
15	Potamogeton malaianus	70% Ethanol	101.2622877
16	Polygonum orientale	70% Ethanol	99.05309459
17	Rorippa palustris	70% Ethanol	98.81111068
18	Artemisia capillaris	70% Ethanol	98.78797193
19	Rorippa palustris	70% Ethanol	97.53363308
20	Ranunculus sceleratus	70% Ethanol	96.85619865
21	Echinochloa crusgalli var. echinatum	70% Ethanol	96.44690059
22	Cardamine flexuosa	70% Ethanol	94.81542257
23	Phalaris arundinacea	70% Ethanol	94.71624117
24	Nymphaea tetragona	70% Ethanol	94.17852836
25	Phacelurus latifolius	70% Ethanol	91.50281441
26	Rorippa palustris	70% Ethanol	91.43114013
27	Polygonum lapathifolium	70% Ethanol	91.00488103
28	Glyceria leptolepis	70% Ethanol	90.94054976
29	Commelina communis	70% Ethanol	90.93825605
30	Carex glabrescens	70% Ethanol	90.42230098
31	Stachys japonica	70% Ethanol	89.21256092
32	Alopecurus aequalis	70% Ethanol	89.17621407
33	Nymphaea tetragona	70% Ethanol	88.56258162
34	Potamogeton distinctus	70% Ethanol	87.05096464
35	Phacelurus latifolius	70% Ethanol	86.62785993
36	Myriophyllum spicatum	70% Ethanol	85.50325667
37	Polygonum maackianum	70% Ethanol	84.77848292
38	Polygonum lapathifolium	70% Ethanol	84.27445606
39	Sparganium erectum	70% Ethanol	83.91986519
40	Eclipta prostrata	70% Ethanol	83.50952422
41	Carex miyabei	70% Ethanol	82.35632603
42	Monochoria korsakowii	70% Ethanol	81.45094041
43	Echinochloa oryzicola	70% Ethanol	80.15221615
44	Carex miyabei	70% Ethanol	79.97735884
45	Rorippa cantoniensis	70% Ethanol	79.14465837
46	Magnolia sieboldii	70% Ethanol	78.84577265
47	Aeschynomene indica	70% Ethanol	78.62087081
48	Phalaris arundinacea	70% Ethanol	78.07080585
49	Rorippa palustris	70% Ethanol	78.05944229
50	Iris pseudacorus	70% Ethanol	77.11935175
51	Nelumbo nucifera	70% Ethanol	77.10078079
52	Ranunculus chinensis	70% Ethanol	76.89069885
53	Scirpus tabernaemontani	70% Ethanol	76.36392406
54	Acorus calamus	70% Ethanol	76.27050326
55	Penthorum chinense	70% Ethanol	75.15182201
56	Impatiens textori	70% Ethanol	74.7126084
57	Lysimachia davurica	70% Ethanol	74.39095995
58	Hydrilla verticillata	70% Ethanol	74.3881919
59	Spiraea prunifolia var. simpliciflora	70% Ethanol	74.04575459
60	Cyperus michelianus var. pacificus	70% Ethanol	73.76067555
61	Polygonum viscosum	70% Ethanol	73.4412207
62	Polygonum thunbergii	70% Ethanol	72.92719876
63	Nymphoides indica	70% Ethanol	72.45421686
64	Artemisia capillaris	70% Ethanol	71.12185913
65	Carex neurocarpa	70% Ethanol	70.83812866
66	Equisetum arvense	70% Ethanol	70.24607777
67	Salix siuzevii	70% Ethanol	69.65646154
68	Lilium lancifolium	70% Ethanol	68.19102445
69	Arthraxon hispidus	70% Ethanol	67.73027076
70	Sium suave	70% Ethanol	67.4776183
71	Aeschynomene indica	70% Ethanol	65.91617813
72	Otteliaismoides	70% Ethanol	65.8915606
73	Juncus effusus var. decipiens	70% Ethanol	65.72563286
74	Salix subfragilis	70% Ethanol	65.17243756

75	Panicum dichotomiflorum	70% Ethanol	64.70117861
76	Lactuca indica	70% Ethanol	64.53310576
77	Potamogeton crispus	70% Ethanol	64.42421065
78	Lilium lancifolium	70% Ethanol	64.39434407
79	Kyllinga brevifolia	70% Ethanol	63.72269127
80	Aster pilosus	70% Ethanol	63.31430046
81	Ribes mandshuricum (Maxim.) Kom.	70% Ethanol	63.2916042
82	Scirpus radicans	70% Ethanol	63.00291949
83	Sicyos angulatus	70% Ethanol	62.83366263
84	Ailanthus altissima	70% Ethanol	62.82969474
85	Rorippa cantoniensis	70% Ethanol	62.59544032
86	Geranium sibiricum	70% Ethanol	62.58701803
87	Oenothera biennis	70% Ethanol	62.5865479
88	Artemisia indica	70% Ethanol	62.5520664
89	Scirpus triqueter	70% Ethanol	62.35934873
90	Iris pseudacorus	70% Ethanol	62.20884179
91	Angelica dahurica	70% Ethanol	62.18227223
92	Morus alba	70% Ethanol	61.5095885
93	Persicaria maackiana	70% Ethanol	61.44179025
94	Ranunculus sceleratus	70% Ethanol	61.11376577
95	Bidens frondosa	70% Ethanol	60.93088491
96	Acer pseudosieboldianum	70% Ethanol	60.44632298
97	Hemistepta lyrata	70% Ethanol	59.67764993
98	Eleocharis mamillata var. cyclocarpa	70% Ethanol	59.50109946
99	Impatiens nolitangere	70% Ethanol	59.33478938
100	Morus alba	70% Ethanol	59.29208944
101	Salix pseudolasiogyne	70% Ethanol	59.27356215
102	Aralia elata	70% Ethanol	59.19285799
103	Leonurus japonicus	70% Ethanol	58.88190471
104	Populus x tomentiglandulosa	70% Ethanol	58.48940487
105	Najas marina	70% Ethanol	58.45009758
106	Salix subfragilis	70% Ethanol	58.39728445
107	Flueggea suffruticosa	70% Ethanol	58.29682068
108	Populus x tomentiglandulosa	70% Ethanol	58.05189502
109	Hydrocotyle maritima	70% Ethanol	57.92963178
110	Stachys japonica	70% Ethanol	57.88630555
111	Rumex crispus	70% Ethanol	57.85553581
112	Phalaris arundinacea	70% Ethanol	57.5376054
113	Arthraxon hispidus	70% Ethanol	57.53732041
114	Impatiens textori	70% Ethanol	57.51341932
115	Echinochloa crusgalli var. echinatum	70% Ethanol	57.37174039
116	Salix chaenomeloides	70% Ethanol	57.25588749
117	Silene armeria	70% Ethanol	57.18705382
118	Arthraxon hispidus	70% Ethanol	56.91037308
119	Salix gracilistyla	70% Ethanol	56.87242675
120	Chelidonium majus var. asiaticum	70% Ethanol	56.64030387
121	Echinochloa oryzicola	70% Ethanol	56.45644973
122	Populus x tomentiglandulosa	70% Ethanol	56.36122086
123	Carex maackii	70% Ethanol	56.30817895
124	Clematis apiifolia	70% Ethanol	56.01472236
125	Leonurus japonicus	70% Ethanol	55.89504815
126	Rosa multiflora	70% Ethanol	55.82072545
127	Fagopyrum esculentum	70% Ethanol	55.76301642
128	Catalpa ovata	70% Ethanol	55.73129832
129	Salix subfragilis	70% Ethanol	55.41917169
130	Euphorbia hypericifolia	70% Ethanol	55.23510636
131	Aeschynomene indica	70% Ethanol	55.03448846
132	Carex pumila	70% Ethanol	54.58343025
133	Cyperus microiria	70% Ethanol	54.50016774
134	Persicaria viscosa	70% Ethanol	54.34371687
135	Scirpus fluviatilis	70% Ethanol	54.29001692
136	Commelina communis	70% Ethanol	54.1231353
137	Penthorum chinense	70% Ethanol	53.92644282
138	Carex miyabei	70% Ethanol	53.87739368
139	Eleocharis ussuriensis	70% Ethanol	53.65875711
140	Pueraria lobata	70% Ethanol	53.24606628
141	Flueggea suffruticosa	70% Ethanol	53.23558254
142	Rosa multiflora	70% Ethanol	53.09636132
143	Salicornia europaea	70% Ethanol	53.05519949
144	Echinochloa crus-galli	70% Ethanol	53.03735713
145	Persicaria perfoliata	70% Ethanol	52.73364752
146	Najas marina	70% Ethanol	52.62521125
147	Persicaria lapathifolia	70% Ethanol	52.32747593
148	Sagittaria sagittifolia var. leucopetala	70% Ethanol	52.26294952
149	Carex miyabei	70% Ethanol	52.12043626
150	Penthorum chinense	70% Ethanol	51.95194152
151	Persicaria thunbergii	70% Ethanol	51.87946213

152	Salix gracilistyla	70% Ethanol	51.58786375
153	Barnardia japonica	70% Ethanol	51.39117365
154	Rumex longifolius	70% Ethanol	51.28465453
155	Salix gracilistyla	70% Ethanol	51.12105966
156	Salix koriyanagi	70% Ethanol	50.92636425
157	Monochoria korsakowii	70% Ethanol	50.83698942
158	Oenanthe javanica	70% Ethanol	50.49257076
159	Medicago sativa	70% Ethanol	50.21112381
160	Salix gracilistyla	70% Ethanol	50.18372808
161	Rumex crispus	70% Ethanol	50.08328056
162	Carduus crispus	70% Ethanol	49.95327969
163	Polygonum viscosum	70% Ethanol	49.48488482
164	Hemistepta lyrata	70% Ethanol	49.38629251
165	Rodgersia podophylla A. Gray	70% Ethanol	48.80641285
166	Salix chaenomeloides	70% Ethanol	48.57690733
167	Echinochloa crusgalli	70% Ethanol	48.55860572
168	Geranium sibiricum	70% Ethanol	48.51027213
169	Acer tataricum subsp. ginnala	70% Ethanol	48.16623629
170	Ampelopsis brevipedunculata	70% Ethanol	48.05972407
171	Iris sanguinea	70% Ethanol	47.99411786
172	Cyperus nipponicus	70% Ethanol	47.85682211
173	Rumex obtusifolius	70% Ethanol	47.84301401
174	Acer tataricum subsp. ginnala	70% Ethanol	47.83713093
175	Salix chaenomeloides	70% Ethanol	47.68638718
176	Schoenoplectiella triangulata	70% Ethanol	47.63070296
177	Salix gracilistyla	70% Ethanol	47.4557175
178	Lythrum anceps	70% Ethanol	46.82113944
179	Cardamine leucantha	70% Ethanol	46.76150681
180	Rumex obtusifolius	70% Ethanol	46.72918204
181	Pueraria lobata	70% Ethanol	46.64832981
182	Scopolia japonica Maxim.	70% Ethanol	46.57878603
183	Potentilla supina	70% Ethanol	46.42721139
184	Polygonum muricatum	70% Ethanol	46.11575408
185	Salix caprea	70% Ethanol	46.00589157
186	Ampelopsis brevipedunculata	70% Ethanol	45.7758842
187	Nymphoides peltata	70% Ethanol	45.69122775
188	Ceratophyllum demersum	70% Ethanol	45.60827736
189	Pueraria lobata	70% Ethanol	45.38221377
190	Persicaria senticosa	70% Ethanol	45.3588724
191	Salix pierotii	70% Ethanol	45.30402545
192	Leonurus japonicus	70% Ethanol	45.19500833
193	Lamium amplexicaule	70% Ethanol	45.18812804
194	Impatiens textori	70% Ethanol	45.15652118
195	Beckmannia syzigachne	70% Ethanol	45.11774124
196	Ailanthus altissima	70% Ethanol	44.98733713
197	Scirpus radicans	70% Ethanol	44.53137709
198	Salix koreensis	70% Ethanol	43.93150135
199	Salix subfragilis	70% Ethanol	43.88107028
200	Bidens pilosa	70% Ethanol	43.86358831
201	Scirpus wichurai var. asiaticus	70% Ethanol	43.60616083
202	Artemisia rubripes	70% Ethanol	43.58422426
203	Betula dahurica	70% Ethanol	43.52871034
204	Hemarthria compressa var. japonica	70% Ethanol	43.42948402
205	Salvia plebeia	70% Ethanol	43.37839048
206	Spiraea prunifolia var. simpliciflora	70% Ethanol	43.37005769
207	Sigesbeckia pubescens	70% Ethanol	43.34140771
208	Lilium lancifolium	70% Ethanol	43.02905526
209	Alnus firma	70% Ethanol	43.00709689
210	Scirpus wichurai var. asiaticus	70% Ethanol	42.95332925
211	Polygonum orientale	70% Ethanol	42.88335212
212	Catalpa ovata	70% Ethanol	42.85563412
213	Platycladus orientalis	70% Ethanol	42.51075544
214	Pueraria lobata	70% Ethanol	42.29175836
215	Salix chaenomeloides	70% Ethanol	42.2239998
216	Metaplexis japonica	70% Ethanol	42.20801684
217	Pinus koraiensis	70% Ethanol	42.17755229
218	Cirsium pendulum	70% Ethanol	42.12158859
219	Potamogeton wrightii	70% Ethanol	42.0709699
220	Sicyos angulatus	70% Ethanol	41.99342268
221	Cyperus difformis	70% Ethanol	41.70672024
222	Salix gracilistyla	70% Ethanol	41.37551623
223	Digitaria ciliaris	70% Ethanol	41.27821485
224	Stachys japonica	70% Ethanol	41.00034688
225	Corydalis pauciovulata	70% Ethanol	40.90604192
226	Lespedeza cuneata	70% Ethanol	40.84278535
227	Setaria faberi	70% Ethanol	40.69826116
228	Salix subfragilis	70% Ethanol	40.65879732

229	Catalpa ovata	70% Ethanol	40.5036856
230	Scirpus tabernaemontani	70% Ethanol	40.48291615
231	Salix koriyanagi	70% Ethanol	40.39490637
232	Rumex crispus	70% Ethanol	40.20119641
233	Ambrosia trifida	70% Ethanol	40.04117491
234	Linderniaceae sp.	70% Ethanol	39.81462732
235	Ipomoea rubriflora	70% Ethanol	39.63257379
236	Salvia plebeia	70% Ethanol	39.56559629
237	Equisetum palustre	70% Ethanol	39.54903493
238	Patrinia scabiosifolia	70% Ethanol	39.49141004
239	Salvia plebeia	70% Ethanol	39.42668692
240	Cyperus glomeratus	70% Ethanol	38.6701162
241	Ostericum grosseserratum	70% Ethanol	37.98013338
242	Carduus crispus	70% Ethanol	37.97573074
243	Salix pierotii	70% Ethanol	37.71809934
244	Lycopus lucidus	70% Ethanol	37.20567944
245	Pinus koraiensis	70% Ethanol	37.02399115
246	Scirpus mitsukurianus	70% Ethanol	36.84653503
247	Erigeron annuus	70% Ethanol	36.80697386
248	Eclipta prostrata	70% Ethanol	36.77265508
249	Alnus japonica	70% Ethanol	36.45881681
250	Carex pumila	70% Ethanol	36.34422236
251	Pueraria lobata	70% Ethanol	36.32486179
252	Cyperus difformis	70% Ethanol	36.22490223
253	Nelumbo nucifera	70% Ethanol	36.01915035
254	Ludwigia epilobioides	70% Ethanol	35.97098761
255	Lysimachia clethroides Duby	70% Ethanol	35.89255537
256	Crotalaria sessiliflora	70% Ethanol	35.64555519
257	Rhododendron mucronulatum	70% Ethanol	35.05732219
258	Schoenoplectiella juncoides	70% Ethanol	34.66896587
259	Oenothera biennis	70% Ethanol	34.35048103
260	Castanopsis sieboldii	70% Ethanol	33.52864811
261	Nymphaea tetragona	70% Ethanol	33.44104592
262	Kummerowia striata	70% Ethanol	33.28036306
263	Catalpa ovata	70% Ethanol	32.80330218
264	Potentilla anemonifolia	70% Ethanol	32.77320184
265	Ranunculus sceleratus	70% Ethanol	32.75865606
266	Juncus decipiens	70% Ethanol	32.74287582
267	Najas minor	70% Ethanol	32.5517306
268	Schoenoplectiella triangulata	70% Ethanol	32.06678987
269	Bidens pilosa	70% Ethanol	31.99756426
270	Rumex crispus	70% Ethanol	31.88304955
271	Ludwigia epilobioides	70% Ethanol	31.7042054
272	Arenaria serpyllifolia	70% Ethanol	31.12910682
273	Polygonum amphibium	70% Ethanol	30.37804236
274	Indigofera kirilowii Maxim. ex Palib.	70% Ethanol	30.04578696
275	Juncus papillosus	70% Ethanol	29.1719954
276	Lemna perpusilla	70% Ethanol	28.97946038
277	Hydrilla verticillata	70% Ethanol	28.88619532
278	Capsella bursa-pastoris	70% Ethanol	28.84865801
279	Lycopus lucidus	70% Ethanol	28.74908051
280	Sparganium erectum	70% Ethanol	28.46454625
281	Penthorum chinense	70% Ethanol	28.26778897
282	Penthorum chinense	70% Ethanol	28.23092675
283	Mentha arvensis var. piperascens	70% Ethanol	27.44313782
284	Eleocharis mamillata var. cyclocarpa	70% Ethanol	27.32076925
285	Angelica polymorpha	70% Ethanol	27.03281237
286	Lindernia dubia	70% Ethanol	26.60446608
287	Artemisia capillaris	70% Ethanol	26.33598515
288	Lycopus lucidus var. hirtus	70% Ethanol	26.3208876
289	Lactuca indica	70% Ethanol	26.24878922
290	Solidago altissima L.	70% Ethanol	26.15470003
291	Ammannia coccinea	70% Ethanol	25.43681373
292	Elaeagnus macrophylla Thunb.	70% Ethanol	25.42691714
293	Boehmeria tricuspis var. paraspicata	70% Ethanol	24.67729782
294	Ambrosia artemisiifolia	70% Ethanol	24.3771329
295	Rumex longifolius	70% Ethanol	23.40516542
296	Commelina communis	70% Ethanol	23.27199964
297	Alnus japonica	70% Ethanol	23.18158916
298	Miscanthus sacchariflorus	70% Ethanol	23.11006345
299	Myriophyllum spicatum	70% Ethanol	22.97337853
300	Sparganium erectum	70% Ethanol	22.91705334
301	Alnus hirsuta	70% Ethanol	22.7773937
302	Alnus hirsuta	70% Ethanol	22.65420816
303	Paulownia coreana Uyeki	70% Ethanol	22.41861802
304	Juglans mandshurica	70% Ethanol	22.15516074
305	Penthorum chinense	70% Ethanol	22.12360854

306	Alnus japonica	70% Ethanol	21.87747668
307	Alnus hirsuta	70% Ethanol	21.58015339
308	Paspalum distichum var. indutum	70% Ethanol	21.55927977
309	Salix koriyanagi	70% Ethanol	21.40649477
310	Penthorum chinense	70% Ethanol	21.0794299
311	Salvia plebeia	70% Ethanol	20.81425629
312	Hydrilla verticillata	70% Ethanol	20.7547669
313	Alnus japonica	70% Ethanol	19.93760498
314	Lobelia chinensis	70% Ethanol	19.81516085
315	Cyperus microiria	70% Ethanol	19.50706775
316	Platycladus orientalis	70% Ethanol	19.38679005
317	Euphorbia hypericifolia	70% Ethanol	18.07650925
318	Cornus officinalis	70% Ethanol	17.77241704
319	Aster pilosus	70% Ethanol	17.54444516
320	Ludwigia epilobioides	70% Ethanol	16.86703439
321	Kummerowia striata	70% Ethanol	16.59264202
322	Persicaria longiseta	70% Ethanol	16.5207965
323	Juglans mandshurica	70% Ethanol	16.35675049
324	Artemisia indica	70% Ethanol	16.21687394

Table S2. Top 10 positive and negative ion mode compounds identified from *Nymphoides peltata* extract with chemical structures.

Mode	Compound Name	Chemical Structure
Positive	Oleamide	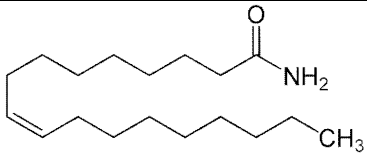
	Hexadecyltrimethylammonium cation	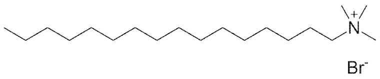
	N-TAmP-FPeSA (N-trimethylammoniopropyl perfluoropropanesulfonamide) (pos)	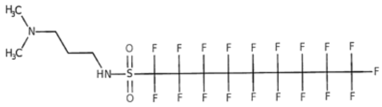
	Oxalic acid mono(N-methyl)amide	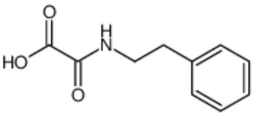
	Amentoflavone	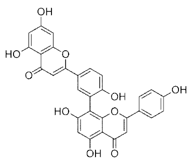
	3-Phenylazopyridine-2,6-diamine	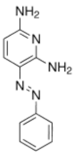
	Peltatoside	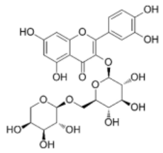
	Tetradonium cation	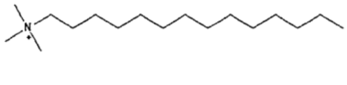
	.alpha.,.beta.-Trehalose	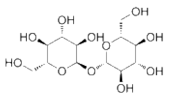
	Bimatoprost	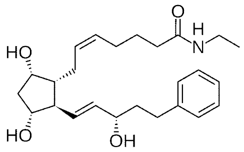

Negative	1,5-DCQA 1,5-Dicaffeoylquinic acid	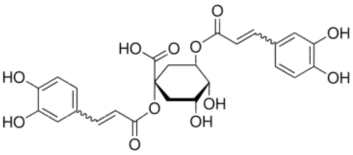
	Ethyl trans-caffeate	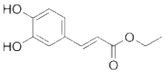
	Peltatoside	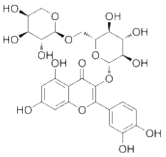
	Chikusetsusaponin IVa	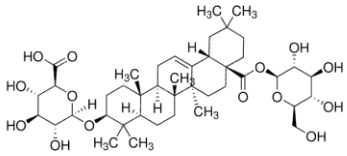
	hesperidin	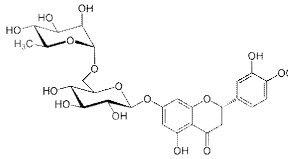
	D-Saccharic acid 1,4-lactone	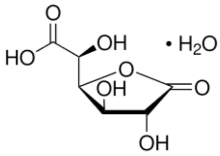
	D-(+)-Trehalose	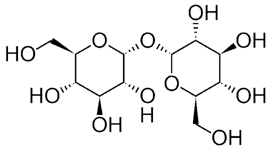
	(+-)-9(10)-Epoxy-12Z-octadecenoic acid	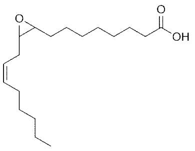
	4-O-Feruloylquinic acid	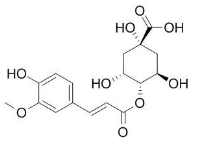
	Melibiose	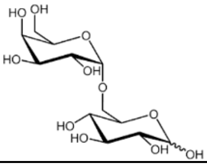
